# Further knowledge and developments in resistance mechanisms to immune checkpoint inhibitors

**DOI:** 10.3389/fimmu.2024.1384121

**Published:** 2024-06-05

**Authors:** Léa Berland, Zeina Gabr, Michelle Chang, Marius Ilié, Véronique Hofman, Guylène Rignol, François Ghiringhelli, Baharia Mograbi, Mohamad Rashidian, Paul Hofman

**Affiliations:** ^1^Inserm U1081 Institute for Research on Cancer and Aging, Nice (IRCAN) Team 4, Université Côte d’Azur, Institut Hospitalo Universitaire (IHU) RespirERA, Federation Hospitalo Universitaire (FHU) OncoAge, Nice, France; ^2^Department of Cancer Immunology and Virology, Dana-Farber Cancer Institute, Boston, MA, United States; ^3^School of Life Science, Ecole Polytechnique Federale de Lausanne (EPFL), Lausanne, Switzerland; ^4^Laboratory of Clinical and Experimental Pathology, Institut Hospitalo Universitaire (IHU) RespirERA, Federation Hospitalo Universitaire (FHU) OncoAge, Pasteur Hospital, Université Côte d’Azur, Nice, France; ^5^Institut Hospitalo Universitaire (IHU) RespirERA, Nice, France; ^6^Hospital-Integrated Biobank (BB-0033–00025), Pasteur Hospital, Nice, France; ^7^Department of Biology and Pathology of Tumors, Georges-Francois Leclerc Cancer Center-UNICANCER, Dijon, France

**Keywords:** cancer, immunotherapy, checkpoint blockade, resistance, biomarkers

## Abstract

The past decade has witnessed a revolution in cancer treatment, shifting from conventional drugs (chemotherapies) towards targeted molecular therapies and immune-based therapies, in particular immune-checkpoint inhibitors (ICIs). These immunotherapies release the host’s immune system against the tumor and have shown unprecedented durable remission for patients with cancers that were thought incurable, such as metastatic melanoma, metastatic renal cell carcinoma (RCC), microsatellite instability (MSI) high colorectal cancer and late stages of non-small cell lung cancer (NSCLC). However, about 80% of the patients fail to respond to these immunotherapies and are therefore left with other less effective and potentially toxic treatments. Identifying and understanding the mechanisms that enable cancerous cells to adapt to and eventually overcome therapy can help circumvent resistance and improve treatment. In this review, we describe the recent discoveries on the onco-immunological processes which govern the tumor microenvironment and their impact on the resistance to PD-1/PD-L1 checkpoint blockade.

## Introduction

The onco-immunology field has witnessed a remarkable boom in the past decade after years of controversial dogmas and inconsistent findings. The upgraded comprehension of the cancer-immune system interactions and the tremendous technological progress have revived the hope of curing cancer with immune-based therapies. The target of these treatments has shifted from the tumor to the host’s immune system, mobilizing immune cells to recognize and eventually eliminate cancer cells. Hallmarks of immunotherapy are the long-lasting response, through immunological memory, and the specificity of a trained immune system to target cancer cells. However, its effectiveness is currently limited to a subset of patients.

ICIs have proven remarkable clinical effects in a wide range of metastatic tumor types. In particular, the PD-1/PD-L1 blocking antibodies act by reactivating pre-existing tumor-infiltrating lymphocytes (TILs) (1). Yost et al. demonstrated that the majority of tumor-specific TILs after anti-PD-1 treatment have TCR specificity not found in the tumor before the therapy, indicating their recruitment post-treatment (2, 3).

Furthermore, a recent scientific investigation has unveiled that innate T cell responses, triggered by ICIs therapies, effectively eliminate tumors by specifically targeting a restricted set of immunodominant neoantigens. The findings of this study also propose that neoTCRs present in polyclonal T cells play a crucial role in generating robust anti-tumor immunity (4).

Independently of their primary immune-related effects, PD-1 and PD-L1 were recently found to induce intrinsic pro-tumoral effects. The expression of PD-1 in melanoma cells has been found to promote tumor growth in immunocompetent as well as in immunocompromised mice (5). Additionally, PD-L1 expression was reported to promote cancer cell survival by conferring resistance to apoptosis induced by T cell cytolytic effectors, cytotoxic drug like staurosporine and interferons (6, 7).

**Figure 1:**
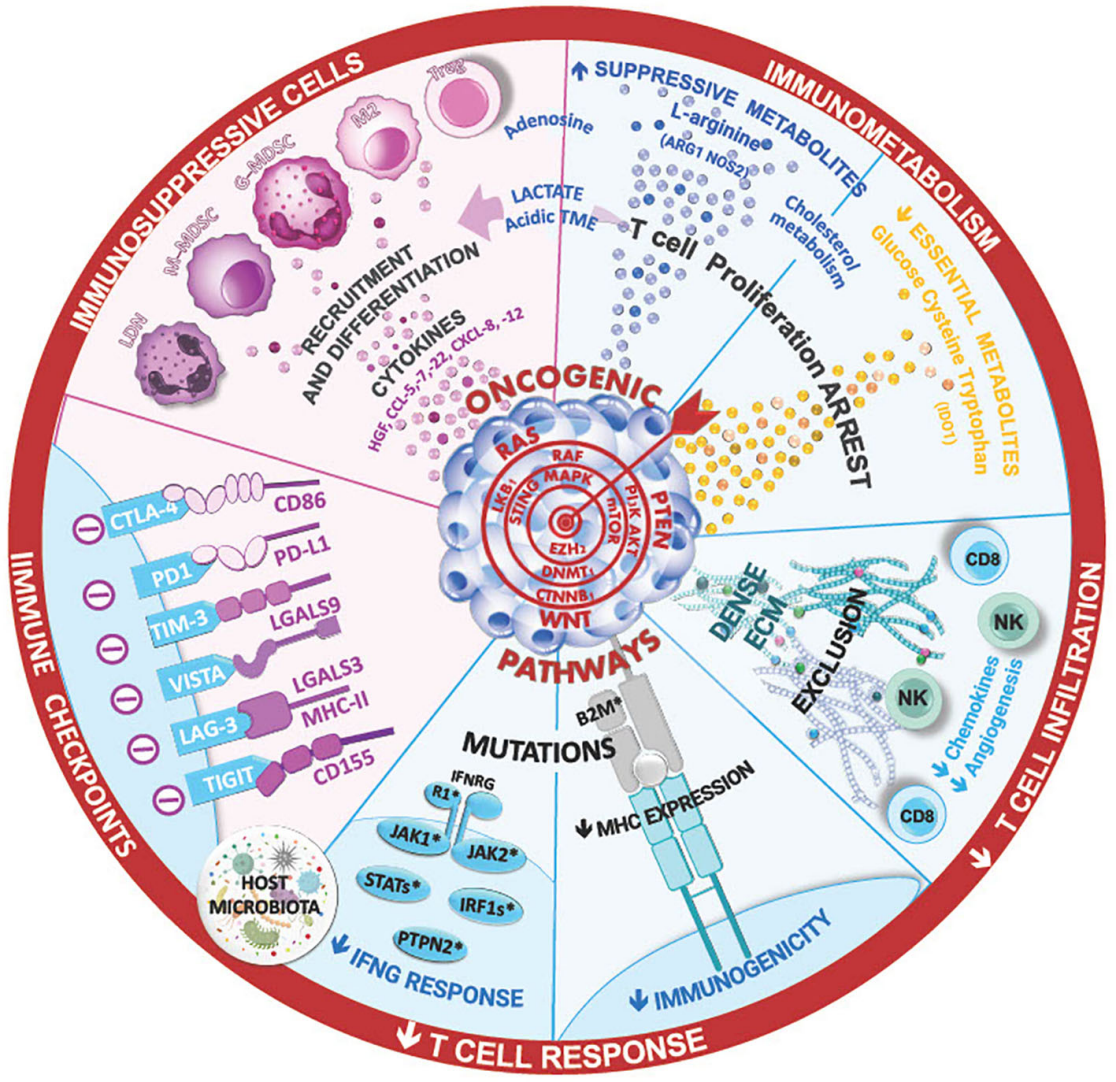
The ineffectiveness of immune checkpoint inhibitor therapies in lung cancer can stem from various mechanism, such as insufficient T-cell infiltration ang high levels of immunosuppressive cells in the tumor microenvironment. Cytokines and the composition of lung and tumor microbial can shape this TME and the immune anti-tumor responses, limiting the efficacy of ICI. Additionally, innate or acquired intrinsic resistance mechanism within cancer cells, such as low tumor mutational burden, mutations in IFN signaling, and antigen presentation pathways, may contribute to treatment resistance. Ongoing research aims to unravel these complexities foir improved therapeutic strategies.

We still lack a comprehensive understanding of the molecular signaling of PD-1 and PD-L1. However, the perspective of using ICIs to reinvigorate the cytotoxic immune responses and concomitantly induce the metabolic reprogramming of tumor cells has made anti-PD-1/PD-L1 immunotherapies even more attractive. Despite the unprecedented durable responses obtained with the anti-PD-1/PD-L1 agents, a large number of patients do not benefit from the treatment (primary resistance) (**Tables 1A**, **1B**), and some responders relapse after a period of response (acquired resistance) (**Table 2**). Moreover, some cancer patients may experience an unexpected acceleration of tumor growth after starting immunotherapy and present with poor outcome in retrospective studies (hyper progressive disease) (**Figure 1**) (**Table 3**) (95, 96).

**Table 1A:** Primary resistance – tumor intrinsic mechanism.

Mechanism	Cause	Consequences	Citation
Genetic mutations	STK11/LKB1 or KEAP1 alterations KRAS-G12D point mutation Mutations B2M or CASP8 gene MATP loss of function	Recruits neutrophils, Inhibits recruitment of T cells, Associated with increased expression of PD-1 and TIM-3 Inhibits CD8+ T cell infiltration Increase PD-L1 level Impaired cell surface expression of MHC class I Defective antigen presentation Lack of CD8 T cells recognition Impaired T cell infiltration and functionality	(8–11) (12) (13–19) (20)
Epigenetic changes	IPRES signature Tumor dedifferentiation Modifications in gene expression of immune-related genes	Upregulation of epithelial to mesenchymal transition, hypoxia, angiogenesis, and wound healing Expression of negative regulatory immune molecules Impact antigen processing, presentation, and tumor immune evasion	(21) (22–25) (26–29)
Alteration in the IFNg signaling pathway	Mutations in IFRNGR1 and IFNGR2, JAK1 and JAK2, IRF-1 and STATs Loss of function in PBAF complex Loss of function of ADAR1	Diminished IFNγ sensitivity, reduced expression of HLA, PD-L1, and anti-tumoral chemokines Facilitates the transcription of IFN-γ-inducible genes leading to the recruitment of T cells and NK cells into the TME Leads to tumor inflammation and growth inhibition	(14, 30–33) (34) (35)
Modification of PD-L1 expression	Oncogenic addiction Inflammatory cytokines PI3K/AKT mutations PTEN deletions EGFR mutations ALK rearrangements MYC overexpression CDK4/CDK6 disruption Increase in PD-L1 transcript	Inhibition of anti-tumor T cell responses	(19, 36–44)
Expression of immuno- suppressive cytokines	TGF-b CCL5, CCL7, CXCL8, CXCL12 or CCL22 CCR1, CXCR2, or CXCR4	Increase of cancer cells invasiveness and promote metastasis Promotion of an immunosuppressive TME through recruitment of MDSCs and Tregs	(45–52) (53–55)

**Table 1B:** Primary resistance – tumor extrinsic mechanism.

Mechanism	Cause	Consequences	Citation
Infiltration of immune suppressor cells	Macrophages Low-density circulating neutrophils	Support neoplastic cell survival, proliferation, angiogenesis, and immune suppression Suppress therapy-induced T cell expansion and effector function	(53, 56–61) (62, 63)
Induction of co-inhibitory molecules expression	Upregulation of CTLA-4, IDO, TIM-3, LAG-3, CD73, and VISTA	Inhibit the function of anti-tumor T cells and dendritic cells	(64–72)

**Table 2:** Secondary resistance mechanism.

Mechanism	Cause	Consequences	Citation
T cell dysfunction	Defects in the antigen presentation machinery of T cells Mutations in the IFNg receptor pathway (JAK1 and JAK2) *De novo* DNA methylation	Failure of T cell activation Tumor escape due to decreased antigen presentation Decreased in T cell infiltration due to lower expression of T cell chemoattractant Irreversible T cell exhaustion	(30) (33) (73, 74)
Changes in the mutational landscape	Low TMB but high intratumor heterogeneity Decreased expression or mutations in tumor neoantigens	Low neoantigens exposure, leading to decreased effector functions Immune escape	(75–77) (78–80)
Induced expression of alternative immune checkpoints	LAG-3, TIGIT, TIM-3 and VISTA re-expression	Promotes immune escape and the suppressive function of MDSCs in the TME	(78–81)
Metabolic alterations	Increased expression of extracellular adenosine Induction of the LXR pathway Warburg effect Hypoxia	Inhibits T-cell proliferation, cytotoxic activity and promotes metastasis Diminish the clonal expansion of T lymphocytes Leads to a Th17 phenotype associated with an inhibition of the anti-tumoral immune response Inhibits the maturation and migration of dendritic cells (DCs) Decrease in ROS production Impair the anti-tumor activity of CD8+ T cells Boost the immunosuppressive cell populations such as MDSCs, TAM, Th2 CD4+ T cells and Tregs	(45, 82–86) (87)
Alterations within the TME	Increased angiogenesis	Decreased number of antitumoral T cells and increased number of TAMs	(88, 89)

**Table 3:** Hyperprogressive disease.

Mechanism	Cause	Consequences	Citation
Genetic alteration	EGFR alteration and MDM2 amplification DNMT3a alteration	Inhibition of p53	(90, 91)
Alteration in oncogenic pathways	Alteration of FGF2/β-catenin oncogene pathway	Escape through a T cell dependant mechanism	(92)
Modification of the immune infiltration	Imbalance between Teff and regulatory T cells (Treg) Increased infiltration of Type 2 macrophages	Escape through a T cell dependant mechanism Activated through the Fc portion of the ICIs	(93) (91, 92, 94)

Thus, it is crucial to address the primary and acquired resistances to ICIs, which emerge as significant clinical challenges. However, it is important to remember that the immune response is constantly evolving and unique to each patient. By understanding the host’s environmental and genomic factors that influence the immune response, we aim to develop more effective treatment interventions, ultimately improving patient outcomes (56).

## Primary resistance to immune checkpoint blockade

Multiple studies demonstrated that a combination of both tumor-intrinsic and tumor-extrinsic factors may contribute to immunotherapy resistance (57). Tumor-intrinsic mechanisms include genetic and epigenetic modifications that prevent the processing and presentation of tumor neoantigen, as well as T cell infiltration or action within the tumor microenvironment (TME) (97, 98) (**Table 1A**). Tumor-extrinsic factors include inadequate T cell function, non-cancerous stromal or immune cells, and other systemic influences that can act with cancer cells to promote resistance to ICIs (57) (**Table 1B**). These mechanisms can either contribute to the primary resistance when detected at the time of the initial diagnosis or highlight the adaptive resistance when detected later during the evolution of cancer under treatment.

### Tumor intrinsic factors

#### Genetic mutations

With each scientific advancement, our comprehension of the fundamental mechanisms governing cancer resistance advances. Attempts to understand the mechanisms of resistance have uncovered that specific genetic mutations can affect the oncogenic signaling, influencing the extent and type of immune infiltration within the TME. Most notably, alterations of *STK11/LKB1* in the presence of *KRAS* mutations have been linked to primary resistance to PD-1 inhibitors in lung adenocarcinoma patients undergoing chemoimmunotherapy (8). The loss of *STK11/LKB1* promotes the production of IL-6, which recruits neutrophils, inhibits recruitment of T cells, and is associated with high levels of T cell exhaustion markers such as PD-1 and TIM-3, and decreased expression of PD-L1 on tumor cells (9, 10). Notably, *KRAS* mutant adenocarcinoma tumors exhibiting LKB1 loss exhibit a significant prevalence of simultaneous *KEAP1* mutations. These mutations activate the KEAP1/NRF2 pathway, a pivotal route in cytoprotection against oxidative stress. This phenomenon contributes to the cancer cells’ ability to resist cytotoxic agents and cytotoxic T cells, enhancing their defense mechanisms against external threats (11, 99). Similarly, the *KRAS-G12D* point mutation has been shown to contribute to an immune-suppressive TME and negatively correlated with CD8^+^ TILs and PD-L1 levels. Specifically, in NSCLC, *KRAS* mutation triggers both the MEK-ERK pathway and the P70S6K/PI3K/AKT pathways, leading to low PD-L1 levels. This also leads to a reduced secretion of the CXCL10 and CXCL11 chemokines by downregulation of HMGA2 signaling, leading to a decrease in CD8^+^ TILS. This results in an immunosuppressive TME, resistant to PD-1/PD-L1 ICIs (12).

*SMARCA4* mutations are detected in 10% of NSCLC cases and are correlated with an immune desert TME, characterized by the absence of tertiary lymphoid structures (TLS) within the TME. Notably, NSCLC with *SMARCA4* mutations exhibits a low response to ICIs, with objective response rates consistently below 20%. A significant proportion of patients also demonstrates minimal infiltration of cytotoxic T cells, while showcasing higher infiltration of pro-tumoral macrophages (100). The current understanding of *SMARCA4* mutations in NSCLC is constrained by a paucity of comprehensive studies and a limited patient cohort available for in-depth analysis. Further complicating matters is the concurrent occurrence of *SMARCA4* mutations with *STK11* and *KEAP1* mutations. The complexity of this molecular interplay makes it challenging to draw conclusions about the impact of *SMARCA4* mutations on the dynamics of immune response and treatment outcomes in NSCLC.

Alternate oncogenic mutations can also hinder the generation of anti-tumor T cells and their exclusion from the TME. This phenomenon has been associated with changes in β-catenin/WNT signaling, a pathway intricately involved in the initiation and progression of various types of cancer. Those modifications lead to reduced CCL4 production and impaired infiltration of CD103^+^ dendritic cells, hampering effective anti-tumor immune responses (13). CD103^+^ dendritic cells secrete CXCL9 and CXCL10 chemokines, crucial components of the anti-tumoral immune response as they attract CXCR3^+^ effector T cells and NK cells. Upon binding to the CXCR3 receptor, CXCL9/CXCL10 chemokines induce effector T cells and NK infiltration to the TME (101).

T cells exclusion from the TME is also associated with loss of function of *CDK2A* and *CDK2B*, two tumor suppressor genes, located at the 9p21 locus and contributing to resistance against immune checkpoint blockade. However, recent investigations by Gjuka et al. have presented an alternative perspective (20). Located at 100 kb from *CDK2A* and *CDK2B*, lies the *MTAP* gene. Gjuka et al.’s research has elucidated that the loss of *MTAP* function is the actual determinant of the deficiency in TILs. The functional impairment of *MTAP* results in the accumulation of methyladenosine (MTA), which detrimentally affects T cell function. MTA promotes the inhibition of protein arginine methyltransferase 5 (PRMT5) and induces activation of adenosine receptor which impedes T cells effector function. Gjuka et al. demonstrated that the administration of MTA-depleting enzymes effectively reinstated TILs infiltration, thereby reducing tumor growth. Furthermore, this intervention synergistically enhanced the efficacy of ICIs, providing empirical validation for the mechanistic association between *MTAP*, MTA accumulation, and T cell dysfunction in the context of immunotherapy resistance.

Moreover, several genomic alterations, such as mutations in beta-2-microglobulin (*β2M*), *JAK1/2* loss of function mutations or *CASP8* gene, which lead to an impaired cell surface expression of MHC class I, defective antigen presentation, and lack of CD8 T cells recognition, have been identified to partially explain the treatment unresponsiveness (14–19).

Finally, aneuploidy, also known as Somatic Copy Number Alteration (SCNAs) is considered one of the main factors driving cancer development, and suspected to be involved in cancer immune evasion. The mechanisms underlying this phenomenon were studied in a comprehensive analysis of 5255 samples from The Cancer Genome Atlas project. This investigation involved examining SCNAs levels and their correlation with the types and number of mutations. Intriguingly, SCNAs levels emerged as a more robust predictor of cytotoxic T cell infiltration than tumor mutational burden (TMB). Additionally, increased SCNAs levels were associated with poorer survival outcomes in patients treated with ICIs, suggesting its potential as a prognostic tool (17).

#### Epigenetic changes

Apart from genetic mutations, resistance to ICIs has also been associated with epigenetic changes, such as the transcriptional IPRES signature (Innate anti-PD-1 Resistance). The IPRES signature consists of the concurrent overexpression of genes involved in the regulation of mesenchymal to epithelial transition, cell adhesion, extracellular matrix remodeling, angiogenesis, and wound healing (21), and was found across various cancer types.

Recent evidence also suggests that tumor dedifferentiation or stemness may also play a role in the resistance to anti-PD-1/PD-L1 blockade. Tumor-initiating stem cells have been found to express negative regulatory immune molecules, such as CD80, PD-L1, and NKG2D (22–25). Interestingly, the β-catenin/WNT signaling, described above in immunotherapy resistance, is also involved in tumor stemness and dedifferentiation (26). Upstream, several epigenetic changes in cancer cells may lead to modifications in gene expression of immune-related genes, which can impact antigen processing, presentation, and tumor immune evasion (56). In pre-clinical studies, this is demonstrated by epigenetic modifying agents, including DNA-methyltransferase inhibitors and histone modifiers. Their mechanism of action involve rescuing the re-expression of components of antigen-processing and presentation machinery, tumor neoantigens, and cytokines, with a potential for therapeutic impact (27–29).

In summary, while checkpoint blockade resistance may stem from a spectrum of genetic and epigenetic modifications, it is essential to recognize that these alterations do not represent the exclusive mechanisms by which tumors evade immune destruction. The dysregulation of immune pathways, exemplified by the interferon-gamma (IFNγ) signaling pathway, emerges as another pivotal factor contributing to the facilitation of tumor immune escape.

#### Alteration in the IFNγ signaling pathway

Critical to the regulation of inflammation and cell-mediated immune responses, mutations within the IFNγ signaling pathway wield a double-edged sword effect in the context of immunotherapy.

Indeed, the sequencing of tumors from patients who did not respond to anti-PD-1 or anti-CTLA4 blockade revealed a high prevalence of loss-of-function mutations in the IFNγ receptor chains (*IFNRGR1, IFNGR2*), the pathway components (*JAK1, JAK2*), the interferon regulatory factor 1 (*IRF1*), the signal transducer and activators of transcription (*STATs*), and the tyrosine-protein phosphatase non-receptor 2 (*Ptpn2*) (14, 30–33). Consequently, upon IFNγ exposure, such mutations would lead to increase expression of PD-L1, leading to cancer cell immunoediting and immune escape (33, 45, 102).

Inversely, mutations in the IFNγ pathway can increase tumor cell sensitivity to ICIs by enhancing the secretion of chemokines that recruit effector T and NK cells to the tumor tissue. For instance, the loss of function of PBAF complex genes (*Pbrm1, Arid2, and Brd7*) can increase the transcription of IFN-γ-inducible genes, increasing the production of effector T cells and NK chemoattractant cytokines (CXCL9/CXCL10) (34). The upregulation of IFNγ expression also leads to an increased expression of the antigen presentation machinery, enhancing cancer cell recognition and facilitating more effective killing. Additionally, reactivation of endogenous retroviral elements and the loss of function of *ADAR1*, an RNA-editing enzyme, could make the cancer more vulnerable to immunotherapy by *viral mimicry* (35).

The dysregulation of the IFNγ pathway is a complex event that can increase sensitivity to immune checkpoint blockade by attracting immune cells and inducing HLA expression on tumor cells. However, it also further exacerbates the evasion tactics employed by cancer cells through PD-L1 overexpression, creating a barrier against the effectiveness of immune-based interventions.

#### PD-L1 expression

Within the TME, PD-L1 is constitutively expressed in response to oncogenic signaling or induced by inflammatory cytokines. Its primary function is to actively inhibit immune anti-tumor T-cell responses. A locus in chromosome 9p24.1 containing the genes for *PD-L1*, *PD-L2*, and *JAK2* is amplified in Hodgkin lymphoma and seems correlated to a high clinical response rate to anti-PD-1 therapy (19).

Co-amplification of *JAK2* and *PD-L1* were also detected in various solid tumors and may be associated with potential valuable metrics in predicting response to immunotherapy (103–107). Other mechanisms that may lead to constitutive PD-L1 expression in tumor cells include *PI3K/AKT* mutations, *PTEN* deletions, *EGFR* mutations, *ALK* rearrangements, *MYC* overexpression, *CDK4/CDK6* disruption, and an increase in PD-L1 transcripts stabilized by truncation of the 3’ UTR of the gene (36–42). In the context of NSCLC, patients with oncogene addiction were frequently excluded from ICIs registration trials. As a result, we have limited clinical knowledge about the efficacy of ICIs in the subgroup of NSCLC patients with oncogene addiction (43). The available data mainly concerns patients with *EGFR* mutation or *ALK* rearrangement, while data for the other less common NSCLC subtypes is lacking. The Immunotarget registry recently demonstrated that ICIs may induce regression in some NSCLC tumors with actionable driver alterations, but clinical activity is significantly lower compared with the *KRAS* group, and the *ALK* group has a notable lack of response (44). Thus, patients with actionable tumor alterations should first receive targeted therapies and chemotherapy before considering immunotherapy as a single agent. Moreover, given the negative impact of the oncogene on the inflammatory TME, a combination of tyrosine kinase inhibitors with ICI may be clinically beneficial for long-term disease control, as recently suggested (43).

### Tumor extrinsic mechanisms

#### Immunosuppressive cytokines

Tumor cells, regulatory T cells (Treg) and M2 macrophages secrete immunosuppressive cytokines to suppress anti-tumor immune responses. Transforming growth factor-β (TGF-β) plays a vital role in immunosuppression by inhibiting the infiltration of cytotoxic T cells through extracellular matrix remodeling (46) and by promoting the activation of Tregs (47–49). Combining anti-TGFβ with anti-CTLA-4 or radiation therapy demonstrated synergistic anti-tumor responses in pre-clinical models (50, 51). TGFβ is also known to induce the expression of transcription factors involved during epithelial-to-mesenchymal transition (EMT) in cancer, such as SNAIL. This process leads to transcription of the Zinc finger protein SNA1 that promotes repression of the E-cadherin cohesion molecule (52). This expression of SNAIL leads to increased production of immunosuppressive molecules such as IL-10 and TSP1 that increase cancer cell invasiveness and metastasis. The expression of these transcription factors also increases the transcription of immunosuppressive elements such as IL-10 and CSF1. Numerous results indicate that the use of TGFβ or TGFβ-related immunosuppressive molecules (IL-10, IL-6, IL-8, VEGF, and CSF1) can be beneficial. Additionally, inhibitors of cells such as TAMs/MDSCs and Tregs may help rescue an immune response to anti-PD-1/PD-L1 immunotherapy (45).

Similarly to TGF-β, certain chemokines (e.g., CCL5, CCL7, CXCL8, CXCL12, or CCL22), along with their corresponding chemokine receptors (e.g., CCR1, CXCR2, or CXCR4) play a significant role in creating an immunosuppressive TME. These are responsible for the attraction of myeloid-derived suppressor cells (MDSCs) and Tregs to the tumor (53, 54). For instance, CCL22 recruits immunosuppressive CCR4^+^ Tregs or CSF1R^+^ macrophages and MDSCs into tumors (55). Furthermore, this intricate interplay between TGF-β, chemokines, and their receptors not only shapes the immunosuppressive milieu within tumors but also bears significant implications for ICIs resistance through the recruitment of immunosuppressive cells.

#### Immune suppressor cells

The impact of the immune system, primarily mediated by T cells, is pivotal in determining the response to checkpoint blockade. However, it is essential to acknowledge that various other immune cell populations also shape the outcomes of immunotherapeutic interventions. Tregs, MDSCs, M2-polarized tumor-associated macrophages, and Th2 CD4^+^ T cells have been linked to ICIs resistance (56, 57). These cells promote an immune suppressive microenvironment that suppresses effector T cell responses through the secretion of cytokines and chemokines or by direct cell contact. In lung cancer, macrophages, pivotal regulators of tumor angiogenesis, secrete growth factors such as VEGF-A and angiopoietin-2. These factors support neoplastic cell survival, angiogenesis, and immune suppression at ectopic sites (58, 59). Many pre-clinical studies have demonstrated that the depletion of those immunosuppressive populations may restore a more robust immune response to cancer, overcoming resistance to ICIs (53, 60, 61). Recent studies have also established a clear link between resistance to checkpoint blockade and the presence of low-density circulating neutrophils (LDN) (62). Elevated blood neutrophil levels are correlated with increased serum hepatocyte growth factor (HGF) concentrations, likely linking these factors to immunotherapeutic resistance. HGF/c-MET signaling mobilizes neutrophils, which acquire immunosuppressive properties in T-cell inflamed tissues. Notably, c-MET+ neutrophils suppress therapy-induced T-cell expansion and effector functions, making the C-MET/neutrophil axis a primary oncogenic driver of ICI resistance (63). It is worth noting that while LDN are associated with immunotherapy resistance in single-therapy scenarios it is not observed when combined with chemotherapy, possibly due to observed neutrophil depletion in the latter case.

Immunosuppressive immune cells significantly contribute to patients’ resistance to immunotherapy. This phenomenon underscores the complexity of immune regulation in the TME and emphasizes the necessity for in-depth scientific investigation to decipher the underlying mechanisms.

#### Induction of co-inhibitory molecule expression

The partial response to immunotherapy has frequently been correlated with the notable upregulation of other inhibitory checkpoints, such as CTLA-4, IDO, TIM-3, LAG-3, CD73, and VISTA, upon PD-(L)1 blockade (64, 65). Indeed, it has been observed that cancer patients who develop recurrent disease after anti-PD-1 treatment have increased TIM-3 expression on T cells (65). Pre-clinical models have demonstrated that the combination of checkpoint blockade using LAG-3+PD-1 and TIM-3+PD-1 led to improved responses (66, 67). Additionally, myeloid- and tumor-cell-derived indoleamine-2,3-dioxygenase (IDO) catabolizes tryptophan to the immune suppressive kynurenine, which can contribute to peripheral tolerance and negatively affects T cell function (68). Other immune suppressive enzymes, such as arginase 1, work in cooperation with the IDO pathway to inhibit the function of dendritic cells (108). Moreover, IFNγ induces the upregulation of IDO and another inhibitory molecule, the carcinoembryonic antigen cell adhesion molecule-1 (CEACAM1) (69, 70). Therapeutic antibodies blocking CEACAM1, and TIM-3 have demonstrated improved anti-tumor immune responses (67, 71, 72).

By identifying both tumor-intrinsic and tumor-extrinsic mechanisms of primary resistance, immuno-oncology has paved the way for multiple lines of attack against cancer. Currently, nearly a thousand clinical trials are testing a combination of anti-PD1 with other therapies. While the precise pathways underlying ICIs resistance have yet to be completely identified, the strong associations between specific axes, signaling pathways, and mutations bring us a step closer to further studies and, ultimately the development of precision immunotherapy.

In conclusion, the challenge of checkpoint blockade resistance is multifaceted. The mechanisms underlying *tumor intrinsic mechanisms of* resistance to checkpoint blockade include genetic and epigenetic alterations, disruption in IFNγ signaling, upregulation of PD-L1 expression, and the influence of immunosuppressive cytokines. Moreover, *tumor extrinsic mechanisms* resistance to ICIs involves adaptive changes within the TME, which poses significant challenges to sustained immunotherapeutic responses. Tumor-extrinsic mechanisms involve non-cancerous stromal or immune suppressive cells, expression of alternate co-inhibitory immune checkpoints, immune suppressive cytokines, or other systemic influences (e.g., host microbiota) that can act in concert with cancer cells to promote resistance to ICIs (97, 109). Altogether, these intricate processes underscore the complexity of tumor immune evasion strategies and emphasize the need for comprehensive research and innovative therapeutic approaches to overcome resistance and enhance the effectiveness of immunotherapy in cancer treatment.

## Acquired resistance to immune checkpoint blockade

While antibodies targeting PD-1 or PD-L1 have shown remarkable and long-lasting clinical effectiveness in some individuals with NSCLC, a significant number of patients who initially respond will eventually experience relapse due to acquired resistance (110). Similarly, it is estimated that one-quarter to one-third of patients with metastatic melanoma who initially respond to anti-PD-1/PD-L1 will experience disease recurrence over time, even when they continue to receive therapy (111). This suggests that the anti-tumor immune response is dynamic, and the mechanisms initially blocked by the treatment tend to turn on inhibitory genes and pathways to tightly regulate immune escape.

Acquired resistance can manifest through various mechanisms, most of which are shared with primary resistance (**Table 2**).

### T cell dysfunction

The primary process of acquired resistance is through T-cell dysfunction. The latter can occur through downregulation of tumor antigen presentation, epigenetic alterations, and acquisition of escape mutations, ultimately leading to T cell exhaustion (56). For instance, a mutation in *β2M* leading to the absence of surface expression of MHC class I was identified in tumor cells from a patient with late acquired resistance to anti-PD-1 treatment (30).

Similar defects in T cell effector functions can lead to acquired resistance to anti-PD-1. In patients with melanoma, anti-PD-1 treatment can induce mutations in the IFNγ receptor pathway, a pathway also prone to disruption in primary resistance. By analyzing melanoma tumor biopsies that relapsed after PD-1 treatment, acquired homozygous loss-of-function mutations were identified in the kinases associated with the interferon-gamma receptor pathway: Janus kinase 1 (JAK1) and Janus kinase 2 (JAK2). Inactivation of *JAK1* and *JAK2* impairs the ability of IFNγ to exert its antitumor effects and renders the tumor unresponsive to anti-PD-L1 (33).

Another mechanism through which patients acquire PD-1 resistance occurs at the T-cell post-effector level. Working with preclinical models, Youngblood et al., have discovered how T cells become exhausted and unable to attack cancer cells as a result of PD-1 treatment. Whole-genome bisulfite sequencing of murine CD8^+^ T cells identified progressive *de novo* methylation programs that restrict their effector function. This provides the rationale of combining ICI with the epigenetic drug decitabine to rescue T cell rejuvenation during PD-1 blockade treatment (73, 74).

### Changes in the mutational landscape

For anti-PD1/PDL-1 therapy to remain effective, the tumor must maintain a sufficient level of immunogenicity. Melanoma is amongst the cancers that are most immunogenic and has one of the highest objective response rates to PD-1 checkpoint blockade (75). Data suggests that anti-tumor T cells activated by checkpoint blockade are specific to tumor antigens presented by the MHC. Those antigens, absent in normal tissues, are called neoantigens. The prevailing understanding in immunotherapy suggests that a higher TMB is a crucial biomarker for identifying cancer patients who are likely to benefit from ICIs. This hypothesis is based on the observed correlation between high TMB and enhanced neoantigen presentation, which amplifies tumor immunogenicity. However, a pre-clinical study conducted on mouse melanoma models found that a higher TMB does not correlate with a better immune checkpoint response. On the other hand, a low intra-tumor heterogeneity (ITH) has been associated with better overall response in immune checkpoint cohorts. This suggests that diminishing the diversity of tumor mutations might make reactive neoantigens more exposed to tumor-infiltrating T cells, leading to a better effector function (75). Therefore, it is not necessarily the increased number of mutations that will lead to a better response but rather the level of diversity of these tumors, with excessive mutational diversity leading to a poor prognosis in immune checkpoint blockade melanoma cohorts.

Additionally, tumors can develop acquired ICIs resistance through decreased expression or mutations in their tumor neoantigens. Over time, this will lead to the killing of immunogenic tumor clones and the growth of the clones harboring poorly immunogenic mutated tumor antigens, leading to immune escape. Consequently, variation of neoantigen level has been proposed as a key mechanism contributing to acquired resistance.

In summary, although tumors with a high clonal neoantigen burden may initially show a favorable response to ICIs and longer progression-free survival, patients may develop acquired resistance to ICIs due to the evolving mutational landscape of tumor neoantigens (76, 77).

### Induced expression of alternative immune checkpoints

Other alternative immune checkpoint molecules may contribute to acquired resistance to PD-1/PD-L1 blockade. LAG-3, TIGIT, TIM-3, and VISTA, four inhibitory checkpoints, are often re-expressed in the TME after an initial response or at the time of relapsed disease (78). Interestingly, hypoxia-induced VISTA promotes the suppressive function of MDSCs in the TME, suggesting that targeting VISTA may mitigate the deleterious effects of hypoxia on anti-tumor immunity (81). Several clinical trials are currently underway to test antibodies against these inhibitory pathways, both as monotherapy and combination therapy strategies (79, 80).

### Metabolic alterations

In addition to the intricate web of immunosuppressive mechanisms within the tumor microenvironment, cancer cells undergo significant metabolic alterations to support their aggressive growth and evade immune surveillance. A key player in promoting immunosuppression is extracellular adenosine. This molecule is produced by the hydrolysis of extracellular AMP, catalyzed by the ectonucleotidases CD39 and CD73. Extracellular adenosine can have diverse implications in anti-tumor immunity, by triggering specific signaling pathways. Specifically, adenosine binding to the A2A receptor inhibits T-cell proliferation and cytotoxic activity (82). Additionally, its engagement with the A2B receptor can promote metastasis, contributing to the development of acquired resistance in cancer (45).

Of interest, the upregulation of CD39 was also shown to suppress CD8^+^ T-cell function and contribute to resistance to PD-1/PD-L1 blockade (83, 84). Thus, co-inhibition of CD39 and PD-L1 could improve anti-tumor immune response and could benefit a large percentage of ICI treated patients (83, 85). Similarly, high levels of soluble CD73 in peripheral blood were associated with a poor response to anti-PD-1 immunotherapy and A2A blockade given concurrently could rescue ICI efficacy (86). Accordingly, both CD39 and CD73 could be used as a potential biomarker of ICI resistance.

Another critical metabolic pathway in the context of tumor-acquired resistance is cholesterol metabolism, which plays a key role in the modulation of the immune response (87). Cholesterol oxidation produces epoxycholesterol and hydroxycholesterol that can bind to the liver X receptor (LXR), leading to its activation. The LXR pathway can diminish the clonal expansion of T lymphocytes, a mechanism that is essential for the activation of these immune cells. In mice, the inhibition of cholesterol esterification by administration of avasimibe, an esterase acetyl-CoA acetyltransferase (ACAT1) inhibitor, enhanced the inhibitory cytotoxic T cells activity [99]. The LXR pathway is essential for the activation but also the polarization of the adaptive immune response. Its activation leads to a Th17 phenotype associated with an inhibition of the anti-tumoral immune response. This pathway also impacts the innate immune response by inhibiting the maturation and migration of DCs, crucial intermediaries bridging the innate and adaptive immunity.

Additionally, cancer cells undergo a metabolic shift known as the Warburg effect, favoring glycolysis and the pentose phosphate pathway over mitochondrial metabolism. This alteration aims to generate ATP and nucleic acids, facilitating rapid proliferation. The lack of mitochondrial activity leads to a decrease of reactive oxygen species (ROSs), protecting tumor cells from cellular damage and promoting their survival.

Cytotoxic T cells are also dependent on the glucose metabolism. Cancer cells impair the anti-tumor activity of CD8^+^ T cells by outcompeting them for glucose consumption. In contrast, T regs rely on fatty acid oxidation (FAO) and remain unimpacted by this competition, enabling them to maintain their immune suppressive activity. Thus, the upregulation of glycolysis and decrease of the amount of ROS produced in cancer cells represents a mechanism of immunosuppression leading to acquired resistance (112).

Furthermore, metabolic abnormalities in the TME are reinforced by poor vascularization. The inadequate formation of blood vessels (vasculature) within the tumor and its surrounding tissue is a hallmark of cancer. This leads to poor supply of oxygen in the TME (hypoxia), making the cancer cells revert to anaerobic glycolysis. As a result, lactate levels are upregulated, which further exacerbates the acidic state of the TME. Low pH boosts the immunosuppressive cell populations such as MDSCs, TAM, Th2 CD4^+^ T cells and Tregs which all have been shown to induce acquired resistance following ICI treatment (88, 89).

### Alterations within the tumor microenvironment

Along this line, the reshaping of the TME following the administration of immunotherapy has been extensively studied. A therapy induced mechanism of resistance was observed in a combination therapy of anti-angiogenic agents and anti PD-1 agents in NSCLC. In cancer, pathological angiogenesis is mediated by the vascular endothelial growth factor A (VEGFA) and angiopoietin-2 (ANGPT2), which both constitute good targets of anti-angiogenic therapies. Their dual inhibition in murine KP and NSCLC mouse models was shown to mediate anti-tumoral effects, through the immune reprogramming of the TME characterized by an increased number of antitumoral T cells and a decrease in TAMs. However, adding PD-1 to that dual inhibition led to relapse (113).

The dual inhibition of angiogenesis was observed to result in the recruitment of PD-1^+^ T regs at a higher proportion than anti-tumoral CD8^+^ T cells. These PD-1^+^ Treg cells were more effectively targeted and activated by anti-PD-1 antibodies. Additionally, intratumoral PD-1^+^ Treg’s were shown to be activated as a result of their interaction with PD-L1^+^ TAMs in murine KP lung tumors. Therefore, within the tumor microenvironment, the infiltration of PD-1^+^ T regs activated by PD-1 antibodies poses an additional obstacle to the efficacy of PD-1 blockade.

To summarize, acquired resistance can arise through a multitude of mechanisms. Those can be grouped into main categories: defects in T cell activation or function, reduced immunogenicity of the tumor, immunosuppression through the reshaping of metabolic pathways or of the tumor microenvironment. Deeper comprehension of fundamental biology holds the potential to enhance therapeutic approaches, allowing to find more precise ways of using and combining immunotherapies in order to circumvent and reverse ICIs acquired resistance.

## Hyper progressive disease

There remains ongoing debate within the scientific community regarding the status of hyper progressive disease (HPD), with divergent opinions regarding whether it represents a distinct pathological entity or merely signifies patients with inherently poor prognostic factors from the onset (114). Cases of patients with advanced cancers, such as NSCLC (13.8%) or head and neck cancer (29%) (115), who experience rapid progression pose serious safety concerns. These cases, identified in 9% of individuals with advanced cancers compared to 2% undergoing targeted therapy, significantly undermine the prospects of success associated with immunotherapy (95, 116). Also observed with PD-1/PD-L1 blockers, hyper progressive disease (HPD) is characterized by accelerated tumor proliferation, high metastatic burden, and early death (mean overall survival of 3.4 months) within the first two months of treatment. HPD is defined as a tumor burden increase of more than 50%, a tumor growth rate exceeding 2-fold, and a time to treatment failure (TTF) of less than 2 months, as outlined in previous studies (95). Although critical, the predictive factors of HPD in patients with cancer treated with anti-PD-1/PD-L1 remain unknown (**Table 3**).

Enhancing our comprehension of HPD is essential for the early identification of susceptible individuals before the initiation of treatment. This understanding is essential in preventing these patients from undergoing potentially detrimental and costly treatment regimens. Identifying HPD early on can facilitate the redirection of these individuals towards alternative therapeutic modalities thereby optimizing the chances of therapeutic success and patient outcomes.

Genomic profiling emerges as a promising avenue for discerning HP disease, as evidenced by a case report study implicating *EGFR* alteration and *MDM2* amplification as potential indicators for HPD in NSCLC) (90, 91). Notably, *MDM2/MDM4* amplification was universally detected in all hyper progressive patients, all experiencing cessation of immunotherapy merely two months post-treatment initiation. Additionally, patients exhibiting *DNMT3a* alteration demonstrated hyper progressive disease in four out of five cases. The concurrent presence of *EGFR* mutation and *MDM2/MDM4* amplification correlated with a TTF of less than two months. *MDM2*, a known inhibitor of *p53*, underpins these observed associations. Further comprehensive investigation is needed to elucidate the intricate molecular mechanisms underlying hyper progressive disease (92, 117).

An integrative study was necessary to gain deeper insights and elucidate the underlying complexities of the mechanisms involved in HPD. Li et al. examined the intricate interplay among immunogenic, metabolic, and oncogenic pathways of cancer patients undergoing immunotherapy (92). Surprisingly, patients who experienced either complete responses (CR) or HPD exhibited similar levels of immune factors, such as IFNγ and CD8^+^ T cell infiltration, as well as comparable T cell clonal diversity. The expression of FoxP3, a T regulatory marker, was also comparable across patients with CR or HPD. While certain gene signatures like *KRAS, NOTCH*, and *EGF* demonstrated similarities, HPD patients displayed an increased activity in pathways associated with *FGF2*, Wnt β-catenin, and stemness invasiveness compared to other groups. These findings were reproducible in several mouse models, including the LLC1 lung adenocarcinoma model, where increased T cell infiltration was evident in HPD cases. Notably, depleting CD8+ T cells resulted in slower tumor growth, suggesting a T cell-dependent mechanism. Further investigation revealed that IFNγ selectively altered NAF^+^/β-catenin signaling in HPD-prone tumor models, confirming the key role of T cells in this mechanism. The disruption of FGF2/β-catenin oncogene pathway was also validated in patients who did not respond to immunotherapy, confirming the study’s clinical relevance.

The role of T effector cells was also confirmed in a study using Near-Infrared Photoimmunotherapy (NIR PIT) (93). NIR PIT is a technology that enables depletion of a specific target population while leaving neighboring cells unaffected. By specifically targeting CD8β, it became possible to deplete effector T cells, leading to an imbalance between Teff and Treg and thereby replicating the immune microenvironment of hyper-progressive tumors. When mice lacking Teff cells were subjected to checkpoint blockade therapy, a significantly accelerated tumor growth was observed compared to the control group lacking Teff cells untreated with checkpoint inhibitors, confirming the key role of CD8^+^ T effector cells in the regulation of HPD.

But T eff cells are not the only component of the immune system playing a crucial role in the development of HPD. In particular, the blockade of PD-1/PD-L1 signaling pathways is known to induce immunosuppression by modulating interactions with innate immune cells. Analysis of pre-treatment tissue samples from patients revealed increased infiltration of Type 2 macrophages within tumors, a phenomenon more pronounced in patients who later exhibited hyper progressive diseases (118). Consistently, murine models also demonstrated enrichment of tumor-associated macrophages within the tumor microenvironment. Notably, in patients, the presence of Type II macrophages expressing the CD163^+^ CD33^+^ PD-L1^+^ phenotype positively correlated with hyper progressive disease, while PD-L1 expression alone showed an inverse correlation. This observation suggests that PD-1 blockade might induce immunosuppression through the interaction of the Fc domain with the inhibitory FcγRIIb receptor, expressed on DCs and monocytes (119). Experimental evidence supporting this notion was derived from athymic nude mice treated with checkpoint blockade, where the removal of the Fc domain from the protein construct led to a decelerated tumor growth rate. Furthermore, administration of nivolumab lacking the Fc domain prevented hyper progressive disease in this model, corroborating the significance of this interaction in the context of immunosuppressive responses (120).

The primary challenge in studying HPD lies in the absence of pre-treatment, as well as during and post-treatment samples. To gain deeper insights, future investigations should focus on collecting tumor and blood samples from HPD patients both before and during treatment. This approach can provide valuable data to elucidate the molecular and cellular mechanisms accelerating disease progression and their direct connection to the treatment process.

## Perspectives

Presently, there is an urgent need to overcome obstacles that hinder the clinical advancements in the field of onco-immunology. These challenges include developing accurate pre-clinical models that mimic human immunity, gaining a comprehensive understanding of the molecular and cellular determinants of primary and secondary resistance, and designing the most effective combinations of personalized immune-based therapies for individual patients (121). Meeting these challenges will require the combined efforts of researchers and clinicians, to accelerate our understanding of the complex interactions between cancer and the immune system, and ultimately develop improved treatment options for cancer patients.

### Combination strategies

In the pipeline, combinatory therapeutic strategies have been explored to target diverse molecular and cellular pathways of resistance (**Table 4**). One established strategy is to combine chemotherapy and immunotherapy. Although counterintuitive at first due to chemotherapy-induced myelosuppression, the chemo- immunotherapy approach has shown significant promise in improving patient outcomes. Chemotherapy inhibits the generation of immunosuppressive immune cells such as T regs, MDSCs, TAMs, thereby promoting a more inflammatory immune infiltrate (122). Additionally, chemotherapy induces tumor cell death, leading to increased presentation of neoantigens (123). A retrospective analysis of NSCLC patients treated with a combination of chemotherapy and immunotherapy demonstrated enhanced overall survival and progression-free survival. Currently, multiple clinical trials (NCT02486718, NCT02657434, NCT02409342, NCT02367781, NCT02366143) are underway to validate the efficacy of Atezolizumab in combination with chemotherapy, aiming to stimulate a robust immune response in NSCLC patients.

**Table 4:** Emerging therapies.

Combination	Registration number	Strategy	Phase
Chemotherapy + Immunotherapy	NCT02486718 NCT02657434 NCT02409342 NCT02367781 NCT02366143	Atezolizumab compared with best supportive care following adjuvant cisplatin-based chemotherapy Atezolizumab in combination with carboplatin or cisplatin + pemetrexed Atezolizumab compared with cisplatin or carboplatin in combination with either pemetrexed or gemcitabine Atezolizumab in combination with carboplatin + nab-paclitaxel Atezolizumab in combination with carboplatin + paclitaxel with or without bevacizumab compared with carboplatin + paclitaxel + bevacizumab	III III III III III
Anti VEGF + Immunotherapy	NCT00790010 NCT05063552	Bevacizumab plus ipilimumab Chemotherapy + cetuximab vs chemotherapy + bevacizumab vs atezolizumab + bevacizumab	I II/III
ACT +/- Immunotherapy	NCT03168438 NCT02992743 NCT02588612 NCT03709706	NY-ESO-1 specific (c259) T cells alone or in combination with pembrolizumab NY-ESO-1c259 T cells Autologous T cells expressing enhanced TCRs specific for NY-ESO-1 Autologous T-Cells expressing enhanced TCRs (T Cell receptors) specific for NY-ESO-1/LAGE-1a alone, or in combination with pembrolizumab	I II I Ib/IIa
Emerging therapies- CAR-T cells	NCT05060796 NCT04153799 NCT03525782 NCT02414269 NCT05693844	CXCR5 Modified EGFR Targeted CAR-T Cells CXCR5 Modified EGFR Targeted CAR-T Cells Anti-MUC1 CAR T Cells and PD-1 Knockout Engineered T Cells Anti-MSLN CAR T Cells CD40 Ligand Expressing MSLN-CAR T Cell Treatment	EarIy phase I I I and II I and II I and II
Emerging therapies - Cytokine therapy + Immunotherapy	NCT02748564 NCT04905316	IL-2 in combination with Pembrolizumab Canakinumab (IL-1β inhibitor) With Chemoradiation and Durvalumab	II I and II

Directing therapeutic efforts toward cancer cells via chemotherapy holds promise, yet the effectiveness of immunotherapy can be increased by direct intervention in the tumor microenvironment. One of the most critical mechanisms in tumor progression is angiogenesis, which fuels nutrients and oxygen to tumor growth. As hoped, integrating anti-VEGF bevacizumab with immunotherapy helps stabilize the tumor vasculature, support the penetration of immune cells and drugs into the tumors and hence boost immunotherapy effectiveness in pre-clinical models (94, 124). Ongoing clinical trials support the potential of this approach in cancer treatment (NCT00790010, NCT05063552).

Checkpoint inhibitors also showed synergistic activity when combined with adoptive cell therapy (ACT). T cells with a transduced TCR can specifically recognize and target cancer cells with high specificity and low toxicity, making them a promising tool in the management of cancer patients. They are currently being investigated in several clinical trials in various cancer types in combination with checkpoint inhibitors such as pembrolizumab or nivolumab (NCT03168438, NCT02992743, NCT02588612, NCT03709706).

Another strategy receiving significant attention is the integration of immunotherapy with cancer vaccines. Therapeutic cancer vaccines are able to enhance the efficacy of ICIs. One approach involves vector-based vaccines like TG4010, which utilize modified viruses encoding specific proteins. In pre-clinical studies, TG4010 has shown significant potential, leading to ongoing phase II clinical trials in combination with ICIs. Another avenue explores dendritic cell-based vaccines like AdCCL21-DC, where genetically modified cells displayed enhanced immune responses. These vaccines have demonstrated encouraging results in animal models, paving the way for phase I clinical trials in patients with advanced cancers.

Exploring diverse combination therapies, including chemotherapy and immunotherapy, anti-angiogenic agents, adoptive cell therapy, and cancer vaccines, offers promising avenues to prevent resistance to ICIs. These innovative approaches, supported by clinical trials, demonstrate the potential to improve cancer immunotherapy, providing patients with more effective and personalized solutions.

### Emerging immunotherapies

More than twenty years after the discovery of the first checkpoint blockade, immunology continues to be the focal point of cancer research, and recent advancements in the past years indicate a promising future. For example, CAR T cells represents a groundbreaking advancement in treating liquid tumors, demonstrating significant efficacy with patients achieving complete remission and experiencing limited toxicities. However, the translation of CAR T cells to solid tumors remains a challenge due to the scarcity of suitable targets. Numerous potential targets for CAR T cell development, including EGFR, HER2, mesothelin (MSLN), prostate stem cell antigen (PSCA), mucin 1 (MUC1), and carcinoembryonic antigen (CEA), among others, have been explored. Nevertheless, only a few have progressed to clinical trials (125).

In a phase I clinical trial assessing the impact of CXCR5-modified CAR T cells targeting EGFR in advanced non-small cell lung carcinoma (NCT05060796), patients exhibited favorable tolerance to the treatment. Subsequent investigations in a second trial (NCT04153799) aimed at optimizing the dosage of EGFR CAR T cell therapy confirmed the low toxicity profile observed in the initial study. However, due to the early stage of these investigations, conclusive remarks regarding the efficacy of anti-EGFR CAR T cells are premature. The need for further exploration will necessitate the initiation of phase II and III clinical trials to comprehensively assess the therapeutic potential of this promising approach.

Exploring an alternative target, a pilot study (NCT03525782) investigates the combined use of MUC1 CAR T cells with PD-1 knock-out T cells, revealing efficacy in primary tumor reduction. However, the findings for metastases present a less encouraging picture.

Mesothelin has also been a focal point of interest as a target for developing CAR T cells designed for solid tumors. However, current clinical trials have not produced promising results, with patients enduring severe toxicities (NCT02414269). Ongoing trials continue to assess the potential toxicities associated with targeting MSLN using CAR T cells. Additionally, investigations are underway to explore the prospect of enhancing CAR T cells through co-expression with CD40L (NCT05693844).

In addition to their associated toxicities, CAR T cells exhibit inherent drawbacks (126). Challenges include the absence of adequate vascularization, downregulation of adhesion molecules, the immunosuppressive tumor microenvironment (TME), and the exhaustion and/or limited infiltration of CAR T cells into the TME, collectively contributing to the observed lack of efficacy. Furthermore, the presence of targeted markers in healthy tissues can lead to aberrant activation of CAR T cells, potentially responsible for their toxicities.

To address these limitations, ongoing research is focused on investigating new markers to enhance the targeting and specificity of CAR T cells for tumors. Promising candidates, such as ephrin-A receptor 2 (EphA2), tissue factor (TF), and protein tyrosine kinase 7 (PTK7), are being explored. These endeavors instill hope that the success achieved by CAR T cells in treating liquid cancers may be replicated in the challenging landscape of solid tumors.

Interleukin therapies have also recently gained prominence due to their promising ability to activate and enhance the cytotoxic capabilities of T cells, including CAR T cells. IL-2, in particular, was the pioneering immunotherapy to exhibit significant antitumor efficacy, with patients demonstrating complete and durable responses in melanoma and renal cell carcinoma. Notably, High-Dose IL-2 (Aldesleukin) stands as the sole interleukin therapy currently approved by the FDA.

Combining HD-IL2 with pembrolizumab holds the potential for a more potent eradication of tumor cells. This combination resulted in partial responses in 11% of treated patients, along with one complete response, underscoring the feasibility and safety of synergizing these two therapies (NCT02748564).

IL-1β is another emerging cytokine of interest. Remarkably, inhibiting its receptor in the context of atherosclerotic disease has shown a reduced incidence of lung cancer. To further explore the potential therapeutic implications, an ongoing phase II clinical trial (NCT04905316) is investigating whether the combination of canakinumab (an anti-IL-1β monoclonal antibody) with chemoradiation and durvalumab proves to be an effective and safe treatment for locally advanced NSCLC. These findings highlight the emerging role of interleukin therapies in enhancing the therapeutic landscape for cancer treatment.

The downside of these therapeutic options lies in the toxicities resulting from on-target or off-site effects. An ideal solution would involve directing the delivery of these drugs specifically to the tumor microenvironment, either through passive or active mechanisms (127).

The principle of passive targeting revolves around delivering drugs through nanocarriers via their passive diffusion or convection through the interstices of tumor capillary pores. Illustrative examples include liposomes, which deliver drugs to the tumor through fusion with the cell membrane, and polymeric nanoparticles (PEG) that enhance drug absorption and blood circulation (128).

Conversely, active targeting entails modifying specific ligands, antibodies, or other molecules on the surface of nanoparticles to identify and attach to particular cells or tissues at the targeted site, ensuring more precise drug delivery. This includes antibody-based targeting, peptide-based targeting, aptamer-based targeting, and small-molecule-based targeting (129, 130).

### Emerging predictive biomarkers

Immunotherapy plays a central role in the treatment of lung cancer and identifying biomarkers that predict response to ICIs (and other immunotherapies) is key. While the predictive power of PD-L1 expression and TMB has long been studied and documented, their accuracy and robustness aren’t consistently reliable. Numerous studies have shed light on emerging biomarkers that can further help with therapeutic response prediction.

The detection of pretreatment PD-L1 protein expression on tumor cells and immune cells by immunohistochemistry is currently the standard practice in the clinical setting. However, it is becoming increasingly clear that PD-L1 remains a controversial biomarker, primarily due to the intratumoral and intertumoral heterogeneity of its expression. Moreover, treatments such as radiotherapy or EGFR tyrosine kinase inhibitor are known to induce changes in its expression levels overtime.

A emerging solution appears with liquid biopsy, allowing the analysis of cancer-related signals in biological fluids. It presents the advantage of being less invasive while being of easier access than tumor biopsies and enabling the analysis of tumor biomolecular features. In a study using liquid biopsies on a cohort of patients, the interest of monitoring the levels of blood PD-L1 and its expression (including PD-L1 mRNA, circulating exosomal PD-L1 and soluble PD-L1) was demonstrated. Blood PD-L1 was shown to have a positive correlation with tumor PD-L1 expression in various malignancies and its upregulation has been correlated with good efficacy and survival for ICIs treatments (131).

Other novel biomarkers are emerging, hoping for better predictor of response than the PD-L1 gold standard. In a retrospective study on a cohort of advanced NSCLC patients, mutations in ARID1A and ARID1B have been proposed as biomarkers for the prognosis and sensitivity to ICI treatment. Deficiencies in those recently discovered oncogenic drivers have been shown to be tightly associated with cancer mutability, PD-L1 expression and are associated with good prognosis for ICIs treatment (132).

Similarly, a recent study identified ZFHX3 mutations as prognostic predictors of NSCLC immunotherapy. Associated with longer overall survival after immunotherapy and demonstrating a positive correlation with other predictive biomarkers such as TMB, ZFHX3 mutations can be used as a novel potential predictive marker to direct NSCLC ICI treatment (133).

Looking at the transcriptome expression profile rather than just the genomics of cancer has also proven to be a valuable tool. Notably, compared to the currently recognized expression of CD274 gene which encodes for PD-L1, the expression of CSF1R and HCST has been shown to have better efficacy in predicting the response to anti-PD-1 therapy in NSCLC. Those genes participates in antigen processing and presentation and T cell receptor signaling pathways, underscoring their significance in this context (134).

Another compelling biomarker is the neutrophil-to-lymphocyte ratio (NLR). It has been extensively studied in recent years as a potential predictive and prognostic tool in patients with NSCLC treated with PD-1/PD-L1 inhibitors. NLR can be used as an inflammation marker and thus has clinical potential in identifying patients that can durably respond to treatment, although prospective studies are needed to confirm its clinical value (135, 136).

Extracellular vesicles (EVs) (which include exosomes and microvesicles) derived from tumor tissues also hold promises as a potential non-invasive biomarker. They play a crucial role in cellular communication by transporting bioactive molecules such as microRNAs, presenting a valuable predictive value. For instance, EV-miR-625–5p has been described as a novel biomarker of response to ICIs in NSCLC patients with PD-L1 expression ≥50% that can thus help stratify them (137).

Microbiota profiling is also increasingly considered a useful tool in predicting response to ICI in NSCLC patients. While an imbalanced respiratory tract microbiome has been associated with tumor progression, a more diverse lung microbiome is correlated with higher levels of CXCL9, a chemokine associated with better immune response in the tumor. More specifically, using 16S RNA sequencing has identified specific microbial enrichments in NSCLC patients with differential ICI responses (138, 139).

Finally, several studies have demonstrated that certain characteristics of the TCR repertoire, such as diversity and density, can influence the effectiveness of immunotherapy in various cancer types. By studying the TCR repertoire before and during treatment, clinicians may be able to identify patients who are more likely to respond to immunotherapy, thereby guiding treatment decisions and improving patient outcomes.

Major developments in TCR sequencing and T-cell antigen specificity prediction have helped with predicting patient outcomes, making it a useful emerging biomarker in the context of cancer (140). As an example, in patients with melanoma, which tend to have a greater T-cell diversity and richness in their peripheral blood and in lymph node metastases, had longer progression-free and overall survival (140). In NSCLC, patients with T cell repertoires that are highly homologous between the tumor and non-involved tumor-adjacent lung showed a lower survival, suggesting that a higher T cell clonality in tumors is correlated with a better prognosis (141).

TCR sequencing characterizes both intratumor as well as intertumoral heterogeneity, which have important implications in explaining mechanisms of cancer immunity and predicting therapeutic responses to immunotherapy. Furthermore, TCR repertoire metrics can also inform about potential immunotherapy-related toxicities. Clonality was assessed in the context of immune-related adverse events (irAEs) after anti-CTLA-4 treatment of prostate cancer patients, showing that the expansion more than 55 CD8+ T-cell clones in the peripheral blood preceded the development of severe irAEs (140).

Thus, there is an ongoing exploration of additional biomarkers, attempting to elucidate why patient responses to immunotherapies differ. Efforts to translate these emergent biomarkers into clinical practice will help strengthen the personalized approach in cancer immunotherapy treatments.

In addition to refining existing therapeutic strategies, it is crucial to enhance patient selection for immunotherapy by excluding individuals who are unlikely to respond or may experience significant side effects. Obtaining tumor tissue before and after treatment initiation is essential for a systematic analysis, enabling a comprehensive understanding of the resistant mechanisms at play (121). Thus, the strategy in identifying the mechanisms of response and resistance to ICIs involves the assessment of serial tumor specimens throughout the course of treatment, together with the development of minimally invasive biomarkers (e.g., liquid biopsy, PBMCs) (56, 142). This approach is important because it encompasses traditional static time points research and aims to recognize superior diagnosis biomarkers by analyzing dynamic responses to ICIs.

## Conclusion

While the revolution of cancer immunotherapy is hurtling down, there is little, if any, time to standardize the companion/complementary tests for routine clinical practice. Whatever the biomarkers and their promise, we are in the rush of their early phases of development; and we require time for global acceptance by large-scale collaborative efforts worldwide (143, 144).

To date, there is no clinically validated biomarker of resistance to ICIs. The onco-immunology research has never been as intriguing, prosper, and promising as nowadays. The revolution of cancer immunotherapies has shed light on a promising decade of success in cancer management, yet large-scale collaborative efforts are crucial to overcoming actual detection, stratification, and resistance obstacles.

Bringing therapeutic benefit to most of patients involves a thorough understanding of the mechanisms that would cause an effective anti-tumor response and the various cell-intrinsic and -extrinsic tumor factors that would give rise to primary, adaptive, and acquired immunotherapy resistance. Elucidating these pathways will provide important insights into the next approaches that need to be taken to effectively resolve immunotherapy resistance.

## Author contributions

LB: Conceptualization, Data curation, Investigation, Methodology, Writing – original draft, Writing – review & editing. ZG: Writing – original draft, Writing – review & editing. MC: Writing – original draft, Writing – review & editing. MI: Writing – original draft, Writing – review & editing. VH: Writing – original draft, Writing – review & editing. GR: Writing – original draft, Writing – review & editing. FG: Writing – original draft, Writing – review & editing. BM: Writing – original draft, Writing – review & editing. MR: Writing – original draft, Writing – review & editing. PH: Writing – original draft, Writing – review & editing.
